# Longitudinal analysis of cerebral aqueduct flow measures: multiple sclerosis flow changes driven by brain atrophy

**DOI:** 10.1186/s12987-020-0172-3

**Published:** 2020-01-31

**Authors:** Dejan Jakimovski, Robert Zivadinov, Bianca Weinstock-Guttman, Niels Bergsland, Michael G. Dwyer, Marcella Maria Lagana

**Affiliations:** 1 Buffalo Neuroimaging Analysis Center (BNAC), Department of Neurology, Jacobs School of Medicine and Biomedical Sciences, University at Buffalo, State University of New York, Buffalo, NY USA; 2 Center for Biomedical Imaging at Clinical Translational Science Institute, University at Buffalo, State University of New York, Buffalo, NY USA; 3 Jacobs Comprehensive MS Treatment and Research Center, Department of Neurology, Jacobs School of Medicine and Biomedical Sciences, University at Buffalo, State University of New York, Buffalo, NY USA; 4 MRI Laboratory, CADiTeR, IRCCS, Fondazione Don Carlo Gnocchi ONLUS, Via Alfonso Capecelatro 66, 20148 Milan, Italy

**Keywords:** Cerebrospinal fluid, Aqueduct of Sylvius, Brain atrophy, Multiple sclerosis, Phase contrast MRI

## Abstract

**Background:**

Several small cross-sectional studies have investigated cerebrospinal fluid (CSF) flow dynamics in multiple sclerosis (MS) patients and have reported mixed results. Currently, there are no longitudinal studies that investigate CSF dynamics in MS patients.

**Objective:**

To determine longitudinal changes in CSF dynamics measured at the level of aqueduct of Sylvius (AoS) in MS patients and matched healthy controls (HCs).

**Materials and methods:**

Forty (40) MS patients and 20 HCs underwent 3T MRI cine phase contrast imaging with velocity-encoded pulse-gated sequence at baseline and 5-year follow-up. For atrophy determination, MS patients underwent additional high-resolution 3D T1-weighted imaging. Measures of AoS cross-sectional area (CSA), average systolic and diastolic velocity peaks, maximal systolic and diastolic velocity peaks and average CSF flow rates were determined. Brain atrophy and ventricular CSF (vCSF) expansion rates were determined. Cross-sectional and longitudinal changes were derived by analysis of covariance (ANCOVA) and paired repeated tests. Confirmatory general linear models were also performed. False discovery rate (FDR)-corrected p-values lower than 0.05 were considered significant.

**Results:**

The MS population demonstrated significant increase in maximal diastolic peak (from 7.23 to 7.86 cm/s, non-adjusted p = 0.037), diastolic peak flow rate (7.76 ml/min to 9.33 ml/min, non-adjusted p = 0.023) and AoS CSA (from 3.12 to 3.69 mm^2^, adjusted p = 0.001). The only differentiator between MS patients and HCs was the greater AoS CSA (3.58 mm^2^ vs. 2.57 mm^2^, age- and sex-adjusted ANCOVA, p = 0.045). The AoS CSA change was associated with vCSF expansion rate (age- and sex-adjusted Spearman’s correlation r = 0.496, p = 0.019) and not with baseline nor change in maximal velocity. The expansion rate of the vCSF space explained an additional 23.8% of variance in change of AoS CSA variance when compared to age and sex alone (R^2^ = 0.273, t = 2.557, standardized β = 0.51, and p = 0.019).

**Conclusion:**

MS patients present with significant longitudinal AoS enlargement, potentially due to regional atrophy changes and ex-vacuo expansion of the aqueduct.

## Introduction

Multiple sclerosis (MS) is chronic inflammatory and neurodegenerative disease of the central nervous system (CNS) characterized with intermittent events of neurological worsening followed by full or partial recovery. In addition to the development of the typical white matter (WM) MS lesions, the disease is also characterized with neurodegenerative and vascular abnormalities [1]. Based on the Monro-Kellie doctrine, the sum of all volumes within the non-elastic cranium have to remain constant. Therefore, hemodynamic changes such as inflammation-driven hyperperfusion or atrophy-driven hypoperfusion will significantly influence the overall brain volume [2]. On the other hand, the continuous neurodegenerative process which leads to high global and central brain atrophy rates will contribute to substantial ex-vacuo enlargement of the ventricular space [3]. Thus, both processes can significantly influence intracranial pressure and thus affect the cerebrospinal fluid (CSF)-derived measures in MS patients. It is currently unknown whether the changes in cerebral fluid dynamics are driven by forces within the CSF space or due to atrophy-driven passive expansion.

A flow-sensitive, magnetic resonance imaging (MRI)-based, phase contrast (PC) cine sequence can serve as a convenient and non-invasive method for quantitative assessment of anatomical and functional aspects of the CSF system [4, 5]. In short, the PC images are generated by subtraction of two dataset which are acquired with bipolar (opposing) gradient sensitizations where the residual image phase shift allows velocity quantification. For best signal quality and to prevent aliasing artifacts, the encoded velocity (V_enc_) should be calibrated approximately to the maximal anticipated flow velocity (in this case, maximal CSF velocity).

Several previous small and cross-sectional studies have investigated CSF flow dynamics in both MS patients and matched healthy controls (HCs) and have reported mixed results [6, 7]. Moreover, a recent MS study also suggested presence of underlying pulse wave encephalopathy which may share multiple pulsation pathophysiological mechanisms similar to the normal pressure hydrocephalus (NPH) [8]. Lastly, in a previous cross-sectional work of ours, we corroborated some presence of slightly altered CSF dynamics in MS patients when compared to HCs [9]. Furthermore, those findings were associated with more severe MRI outcomes including greater lesion volume and smaller central and WM volumes [9]. However, due to the limitations of cross-sectional design, all aforementioned studies were not equipped to provide insights regarding consequential relationships between changes in the CSF fluid dynamics and other MS-related MRI measures.

Until now, there are no reports which have longitudinally analyzed the changes of CSF measures in MS patients and HCs. Based on this background, we aimed to assess longitudinally the changes within AoS-derived variables including its size, average and peak CSF velocity and total CSF flow in both MS patients and age- and sex-matched HCs over a follow-up period of approximately 5 years.

## Materials and methods

### Study population

The population used for this particular analysis was derived from a larger, longitudinal cardiovascular, environmental and genetic study in MS (CEG-MS) [10, 11]. The inclusion criteria for MS patients consisted of: (1) age between 18 and 75 years old at baseline; (2) availability of an anatomical MRI scan with an additional cine PC imaging protocol both at baseline and 5-year follow-up visits; (3) being MS as diagnosed per the 2010-revised McDonald criteria [12]. On the other hand, the exclusion criteria included (1) no clinically-defined relapse or use of intravenous corticosteroid within 30 days of the MRI examination, (2) pregnant or nursing mothers, and (3) presence of congenital malformations that affect the cerebrospinal fluid anatomy (ex. Chiari malformations, congenital hydrocephalus). The inclusion criteria for the HCs was: (1) age between 18 and 75 years old at baseline and (2) no presence of current nor history of past major neurological disorder.

The MS population underwent full clinical evaluation by experienced neurologist and was characterized by the Expanded Disability Status Scale (EDSS) scores [13]. Based on the clinical and radiological presentation, the patients were further classified in relapsing-remitting MS (RRMS) and progressive MS (PMS) subgroups.

### MRI acquisition and analysis

Both MS patients and HCs underwent 3T MRI examination performed at baseline and 5-year follow-up on 3T GE Signa Excite HD 12 Twin Speed 8-channel scanner (General Electric, Milwaukee, WI, USA) and an 8-channel head and neck coil. There were no substantial hardware nor software changes over the follow-up period.

For CSF flow and velocity measurement, a 2D cine PC imaging with velocity-encoded pulse-gated sequence was acquired, perpendicularly to the AoS, with the following parameters: echo time (TE) of 7.9 ms and repetition time (TR) of 40 ms, 20 cm/s velocity encoding, slice thickness of 2 mm, flip angle (FLIP) of 20°, and pulse oximeter-gated procedure for obtaining 32 points of the cardiac cycle. In order to minimize potential flow variations due to different cranio-caudal levels or different head rotations, the acquisition protocol was standardized and the technician paid attention to avoid head tilting/rotation. The imaging plane was positioned at the level of the ampulla, perpendicularly to the AoS axis, visible on the midsagittal scout [14]. For brain volume measurements, MS patients underwent additional high-resolution 3D T1-weighted imaging with a spoiled gradient echo with inversion recovery preparation pulse with TE/inversion time (TI)/TR of 2.8 ms/900 ms/5.9 ms, FLIP of 10°, field of view (FOV) of 25.6 cm × 19.2 cm, voxel size of 1 × 1 × 1mm^3^ with no gaps.

**Fig. 1:**
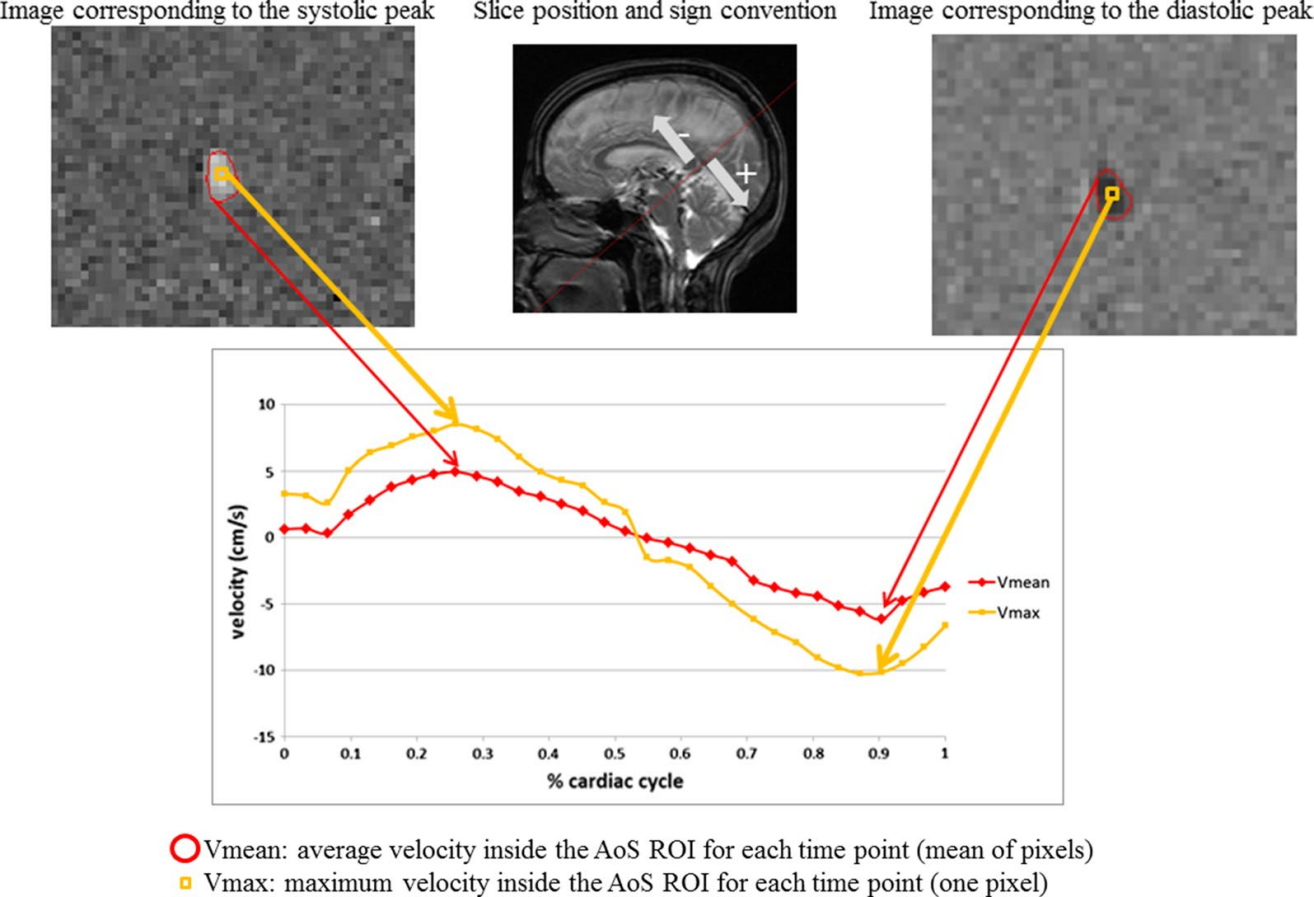
Cross-sectional area of Aqueduct of Sylvius and velocity measures. *AoS* Aqueduct of Sylvius, *ROI* region of interest, *V* velocity

For analysis of the PC cine imaging, freely available software Segment version 2.0 (MedViso, Lund, Sweden) was utilized [15]. The AoS-derived cross-sectional area expressed in mm^2^ (CSA) and velocity measures expressed in cm/s for each pixel inside the CSA contours were derived for all 32 points of the cardiac cycle (Fig. 1). Using these measures were further computed: (1) average area of the AoS, (2) averaged velocity (considering all the pixel inside the segmented CSA; Vmean) for both systolic and diastolic peaks, (3) maximal velocity (pixel with highest velocity inside the segmented CSA; Vmax) for both systolic and diastolic peaks, (4) average CSF flow rate (Vmean*AoS CSA) and (5) systolic and diastolic flow volumes. On the other hand, to determine the rate of brain atrophy within the MS patients, the 3D T1-WI images were inpainted and then analyzed by Structural Image Evaluation using Normalisation of Atrophy (SIENA) and SIENAX multi-time point algorithm, where whole brain (WB) atrophy and ventricular CSF (vCSF) space expansion were calculated, respectively [16]. T1 and T2 lesions and their volume were segmented using validated semi-automated, contouring/thresholding procedure with Java Image Manipulation (JIM, Xinapse systems, Essex, UK) software. In short, SIENA software allows quantitative temporal brain atrophy measurement by utilizing two MRI images from different time points and producing a third “change” image. The images are coregistered to each other, scaled and skewed based on the skull input. Similarly, SIENAX produces intra-subject comparable volumes by using brain-and-skull based correction and scaling into standard MR space. SIENA and SIENAX demonstrate accuracy of 0.2% and 1% error in normalized brain volume analysis [17]. Furthermore, the thalamic atrophy was determined with FMRIB’s Integrated Registration and Segmentation Tool (FIRST) software (FMRIB, Oxford, UK). The volumes of T2 lesion, WB, vCSF and thalamus are expressed in milliliters (ml) and in total and annualized longitudinal % change, for baseline and longitudinal measures, respectively.

### Statistical analysis

All statistical analyses were performed in SPSS 25.0 (IBM, Armonk, USA). The distribution of the data and their residuals was determined by Kolmogorov-Smirnov test for normality and by visual inspection of Q–Q plots. The demographic and clinical differences between MS and HCs were derived by parametric (χ^2^ test, Student’s t-test and analysis of covariance; ANCOVA) and non-parametric (Mann–Whitney U-test) comparisons tests, accordingly. The longitudinal change in AoS variables was determined by appropriate paired repeated measure tests. Lastly, repeated measure analysis within a general linear model (GLM) framework determined the significant effects and associations between the AoS variables and suspected study covariates (age, sex, disease phenotype, rate of global brain atrophy). Confirmatory regression analysis and partial Spearman’s ranked correlations were performed. The aforementioned analyses were separately performed in the RRMS and PMS subtypes as well. The results were corrected for false discovery rate (FDR) utilizing Benjamini-Hochberg procedure. Accordingly, p-values and multiple comparison-corrected q-values < 0.05 were considered statistically significant.

## Results

### Demographic and clinical characteristics

The demographic and clinical characteristics of the study population are shown in Table 1. Similarly, the specifics of the RRMS and PMS subgroups are also shown in Table 1. There were no differences between the MS patients and HCs in age (51.6 vs. 47.4 years old, t-test p = 0.146) nor in female/male ratio (24/16 vs. 15/5, χ^2^ test p = 0.39). Similarly, there were no differences in the follow-up time between the MS patients and HCs (5.6 ± 0.6 vs. 5.5 ± 0.5 years, t-test p = 0.858). The MS population had an average disease duration of 16.2 years. Similarly, the median disability scores were EDSS 2.5 at both baseline and follow-up. The MS population consisted of 26 RRMS and 14 PMS patients. At baseline, 12 (30%) MS patients were on interferon-β, 12 (30%) on glatiramer acetate, 7 (17.5%) on natalizumab, and 9 (22.5%) did not use any disease modifying treatment (DMT). During the 5-year follow-up, 29 (72.5%) MS patients remained on the same DMT regiment, whereas 11 (27.5%) patients have changed their medications. There were no differences in DMT regiment changes between the RRMS and PMS groups (χ^2^ test p = 1.000) Lastly, the total MS population had an average of 13.8% increase in total T2 lesion volume, 2.6% WB atrophy rate and 13.7% of vCSF space expansion rate.

**Table 1:** Demographic and clinical characteristics of the study population

	MS (n = 40)	RRMS (n = 26)	PMS (n = 14)	HCs (n = 20)	MS vs. HCs p-value	RRMS vs. PMS p-value
Female, n (%)	24 (60)	15 (57.7)	9 (64.3)	15 (75)	0.39	0.746
Age, mean ± SD	51.6 ± 9.1	48.8 ± 9.7	56.7 ± 4.9	47.4 ± 12.9	0.146	*0.002*
Follow-up period, mean ± SD	5.6 ± 0.6	5.5 ± 0.6	5.7 ± 0.6	5.5 ± 0.5	0.858	0.423
Disease duration, mean ± SD	16.2 ± 10.6	12.2 ± 9.0	23.6 ± 9.6	–	–	*<* *0.001*
EDSS at BL, median (IQR)	2.5 (1.5–4.75)	1.5 (1.5–2.5)	5.5 (3.4–6.5)	–	–	*<* *0.001*
EDSS at FU, median (IQR)	2.5 (1.5–6.0)	2.0 (1.5–3.0)	6.0 (4.0–6.5)	–		*<* *0.001*
DMT use at baseline, n (%)						
Interferon-β	12 (30)	10 (38.4)	2 (21.4)	–	–	0.51
Glatiramer acetate	12 (30)	6 (23.1)	6 (42.9)	–		
Natalizumab	7 (17.5)	4 (15.4)	3 (21.4)	–		
No DMT	9 (22.5)	6 (23.1)	3 (21.4)	–		
T2 LV change, mean ± SD	13.8 ± 34.4	20.5 ± 39.7	0.9 ± 14.6	–		0.095
Annualized T2 LV change, mean ± SD	2.5 ± 6.6	3.8 ± 7.7	0.2 ± 2.6	–	–	0.112
WBV loss, mean ± SD	− 2.6 ± 1.5	− 2.6 ± 1.4	− 2.5 ± 1.5	–	–	0.831
Annualized WBV loss, mean ± SD	− 0.43 ± 0.22	− 0.48 ± 0.27	− 0.46 ± 0.27	–	–	0.779
vCSF expansion, mean ± SD	13.7 ± 10.5	12.5 ± 9.9	15.8 ± 11.6	–	–	0.384
Annualized vCSF expansion, mean ± SD	2.29 ± 1.8	2.26 ± 1.8	2.89 ± 2.1	–	–	0.353
Thalamus atrophy, mean ± SD	− 4.7% ± 4.7	− 4.5% ± 5.0	− 5.1%4.3	–	–	0.701
Annualized thalamus atrophy, mean ± SD	− 0.85 ± 0.88	− 0.81 ± 0.9	− 0.92 ± 0.8	–	–	0.724

The T2-LV, WBV, vCSF and thalamic volume changes are expressed in longitudinal % change. The lesion and brain volumes are measured as milliliters (mL)

*MS* multiple sclerosis, *RRMS* relapsing-remitting multiple sclerosis, *PMS* progressive multiple sclerosis, *HCs* healthy controls, *EDSS* Expanded Disability Status Scale, *DMT* disease modifying treatment, *LV* lesion volume, *WBV* whole brain volume, *vCSF* ventricular cerebrospinal fluid, *SD* standard deviation, *IQR* interquartile range, *BL* baseline, *FU* follow-up

χ^2^ and Student’s t-test were used. p-value lower than 0.05 was considered statistically significant and shown in italics

As expected, the PMS group was older (56.7 vs. 48.8 years old, t-test p = 0.002), had longer disease duration (23.6 vs. 12.2 years, t-test p < 0.001) and had greater EDSS scores at both baseline and follow-up (both Mann Whitney U-test p < 0.001) when compared to the RRMS group. Similarly to the total group, there were no differences in the follow-up time between the RRMS and PMS patients (5.5 ± 0.6 vs. 5.7 ± 0.6 years, t-test p = 0.423) No differences in female/male ratio (15/11 vs. 9/5 χ^2^ p = 0.746) nor in DMT use (χ^2^ p = 0.51) were noted. Lastly, there were no significant differences in total longitudinal T2 lesion volume (0.9% vs. 20.5%, t-test p = 0.095) nor annualized % change (0.2% vs. 3.8%, t-test p = 0.321), total nor annualized WB atrophy (− 2.5% vs. − 2.6%, t-test p = 0.831 and − 0.46% vs. − 0.48%, t-test p = 0.779), total nor annualized vCSF expansion rate (15.8% vs. 12.5%, t-test p = 0.384 and 2.89% vs. 2.26%, t-test p = 0.353) and total nor annualized thalamic atrophy (− 4.7% vs. 5.1%, t-test p = 0.701 and − 0.92% vs. − 0.8, t-test p = 0.724).

### Phase contrast cine imaging variables

Full description of the cross-sectional and longitudinal change of the AoS-derived variables at baseline and follow-up visits for both the MS and HCs populations are shown in Tables 2 and 3. Due to low phase signal quality of the scan, 5 HCs and 5 MS patients were excluded at the baseline visit, whereas 5 HCs and 9 MS patients were excluded at the follow-up visit. In paired repeated measure analysis, the HCs demonstrated only significant increase in the maximal diastolic peak (from 6.31 to 7.61 cm/s, paired test p = 0.035). On the other hand, the MS population demonstrated significant increase within the maximal diastolic peak (from 7.23 to 7.86 cm/s, paired test p = 0.037), diastolic peak flow rate (7.76 ml/min to 9.33 ml/min, paired test p = 0.023) and in AoS CSA (from 3.12 to 3.69 mm^2^, paired test p = 0.001). Only the latter significant change survived FDR adjustment for multiple comparisons. In terms of between-group comparisons, at baseline, there were no statistical differences in any of the AoS-derived variables between MS patients and HCs. In line with the longitudinal changes, the only differentiator between MS patients and HCs was the greater AoS CSA measured at follow-up (3.58 mm^2^ vs. 2.57 mm^2^, age- and sex-adjusted ANCOVA, p = 0.045).

**Table 2:** Longitudinal analysis of phase contrast flow measures in both MS patients and HCs

AoS-derived measures	HCs (n = 13)			MS (n = 29)		
	Baseline	Follow-up	BL to FU p-value	Baseline	Follow-up	BL to FU p-value
Vmean systolic peak (cm/s)	5.10 ± 2.31	4.89 ± 2.1	0.579	5.46 ± 2.16	5.43 ± 1.73	0.939
Vmean diastolic peak (cm/s)	3.75 ± 2.28	4.14 ± 2.37	0.204	4.22 ± 1.32	4.06 ± 1.20	0.415
Vmax systolic peak (cm/s)	9.16 ± 4.64	9.88 ± 5.03	0.177	9.85 ± 3.56	9.54 ± 2.97	0.53
Vmax diastolic peak (cm/s)	6.31 ± 3.19	7.61 ± 4.35	***0.035***	7.23 ± 2.00	7.86 ± 2.76	***0.037***
Average area (mm^2^)	2.62 ± 1.20	2.86 ± 1.26	0.19	3.12 ± 1.07	3.69 ± 1.55	0.001*
Flow rate systolic peak (ml/min)	8.65 ± 6.05	8.78 ± 5.12	0.882	10.36 ± 5.53	12.16 ± 7.30	*0.051*
Flow rate diastolic peak (ml/min)	6.57 ± 5.59	7.55 ± 5.72	0.207	7.76 ± 3.51	9.33 ± 5.64	***0.023***
Net vol caudal (µl/beat)	37.74 ± 25.34	33.29 ± 19.41	0.334	46.93 ± 27.31	50.49 ± 34.4	0.391
Net vol cranial (µl/beat)	37.89 ± 36.75	38.92 ± 38.5	0.885	41.13 ± 23.56	45.86 ± 32.22	0.208

*HCs* healthy controls, *MS* multiple sclerosis, *AoS* Aqueduct of Sylvius, *Vmean* average velocity, *Vmax* maximal velocity, *BL* baseline, *FU* follow-up

All measures are shown as mean ± standard deviation. Paired repeated measure analysis was utilized. p-values lower than 0.05 were considered statistically significant and shown in bold italics where trending values are shown in italics

* Statistically significant after false discovery rate correction

**Table 3:** Comparison of phase contrast flow measures between MS patients and HCs

AoS-derived measures	At baseline			At follow-up		
	HCs (n = 15)	MS (n = 35)	p-value HCs vs MS	HCs (n = 15)	MS (n = 31)	p-value HCs vs. MS
Vmean systolic peak (cm/s)	5.07 ± 2.29	5.73 ± 2.35	0.611	4.70 ± 1.91	5.40 ± 1.68	0.222
Vmean diastolic peak (cm/s)	− 3.95 ± 2.31	− 4.30 ± 1.45	0.639	− 3.71 ± 2.21	− 4.09 ± 1.16	0.408
Vmax systolic peak (cm/s)	9.08 ± 4.5	9.82 ± 3.48	0.817	9.22 ± 4.74	9.53 ± 2.87	0.834
Vmax diastolic peak (cm/s)	− 6.5 ± 3.16	− 7.32 ± 2.13	0.468	− 6.76 ± 4.1	− 7.82 ± 2.68	0.315
Average area (mm^2^)	2.53 ± 1.14	3.13 ± 1.18	0.325	2.57 ± 1.27	3.58 ± 1.55	***0.045***
Flow rate systolic peak (ml/min)	8.35 ± 5.85	10.61 ± 5.41	0.508	7.71 ± 5.02	11.75 ± 7.22	*0.072*
Flow rate diastolic peak (ml/min)	− 6.60 ± 5.38	− 7.92 ± 3.58	0.582	− 6.32 ± 5.49	− 9.10 ± 5.52	0.132
Net vol caudal (µl/beat)	37.01 ± 24.48	47.42 ± 26.23	0.534	29.59 ± 18.85	49.25 ± 33.63	*0.052*
Net vol cranial (µl/beat)	− 36.91 ± 34.83	− 40.7 ± 23.39	0.895	− 32.49 ± 35.67	− 44.58 ± 31.59	0.302

*HCs* healthy controls, *MS* multiple sclerosis, *AoS* Aqueduct of Sylvius, *Vmean* average velocity, *Vmax* maximal velocity

All measures are shown as mean ± standard deviation. Analysis of covariance (ANCOVA) adjusted for age and sex was utilized. p-values lower than 0.05 were considered statistically significant and shown in bold italics where trending values are shown in italics

Moreover, the longitudinal change and differences of AoS-derived measures within and between the RRMS and PMS patients are shown in Additional file 1: Tables S1, S2. The increase of the AoS size over the follow-up was mainly driven by the increase seen within the PMS patients (from 3.24 to 3.96 mm^2^, paired test p = 0.004). This finding did survive FDR correction. Similarly the PMS group had greater annualized AoS CSA % change when compared to the RRMS (6.3% vs. 1.1%, t-test p = 0.023). No significant differences in AoS-derived measures between the RRMS and PMS subgroups were noted.

### Longitudinal changes in Aqueduct of Sylvius measures and their associations with brain volumes

In order to determine whether changes in AoS CSA were driven by increased CSF velocities or by local atrophy changes, we performed repeated measure GLM models. After adjusting for age and sex, the AoS CSA repeated measure factor had a trending interaction with the longitudinal vCSF expansion rate (Wilks’ Lambda test F = 4.124, partial η^2^ = 0.171, p = 0.056) but not with the baseline nor change in maximal velocity. In confirmatory analysis, the AoS CSA change was associated with vCSF expansion rate (age- and sex-adjusted Spearman’s correlation r = 0.496, p = 0.019) and not with the baseline nor change in maximal velocity. After correcting for age and sex effects, the expansion rate of the vCSF space provided additional 23.8% of explained AoS CSA variance (R^2^ = 0.273, t = 2.557, standardized β = 0.51, and p = 0.019). Lastly, greater AoS CSA change was also associated with more thalamic atrophy in the PMS subgroup (r = − 0.663, p = 0.036).

## Discussion

This study demonstrates that MS patients exhibit significant enlargement in their AoS over mid-term follow-up period. These changes were greater when compared to the HC population and were not accompanied by significant changes in other flow-derived CSF measures. Furthermore, the dilatation of AoS was associated with the rate of global ventricular expansion which is commonly used as a proxy for central brain atrophy in MS patients. These findings were corroborated by associations of thalamic atrophy and dilatation of the bordering AoS.

The complex and multifaceted disease-derived changes that occur within MS patients can influence the CSF dynamics. Based on the aforementioned Monro-Kellie doctrine, the recent findings of anatomical and functional cerebrovascular changes should also change the CSF parameters [18]. For example, the increased intracranial arterial volume (hyperperfusion) during acute inflammation would increase the overall brain volume and result with greater CSF velocities (greater amount of CSF would be propelled out of the cranium) [19]. On the other hand, MS patients also experience chronic narrowing of the major arterial vessels, decreased total amount of cerebral arterial blood flow, and cerebral hypoperfusion [20, 21]. Similar findings of decreased arterial blood flow were seen in a recent PC study which examined all three arterial, venous and CSF compartments [22]. The same study additionally showed lower CSF oscillations in the 19 MS patients when compared to 21 HCs [22]. Phase shifts during cardiac cycle seen in the arteriovenous compartment can directly influence the CSF pulsatility [23]. Such features can be seen in patients of idiopathic intracranial hypertension, where a small increase in arteriovenous pulsations resulted in greater CSF flow through the foramen magnum and increase in intracranial pressure [23]. Moreover, a decrease or loss in cerebrovascular reactivity function will result in the inability to adjust during changes in systemic arterial pressure and allow direct propagation of the pressure pulse into the CSF. This inability to dampen the pressure wave may contribute to greater CSF fluid velocities and oscillations [24, 25]. MS patients may also display increased age- and atherosclerosis-driven arterial stiffening, which can further contribute towards extending the systolic pulse wave into the cerebral arterial vasculature [26]. In our MS sample, we were not able to demonstrate significant differences in CSF velocities when compared to the HCs nor significant changes after 5-year follow-up. Therefore, it does not support the notion of abnormal CSF dynamics within MS patients. Previous and similarly powered studies have shown largely discrepant results. A study which investigated 16 RRMS patients and 8 HCs has demonstrated decreased net CSF flow [27]. Analogous findings of decreased net CSF flow were later demonstrated in a cohort of 67 MS patients and 9 clinically isolated syndrome patients when compared to 35 HCs [9]. Contrarily, another study did not demonstrate differences in net CSF flow nor in CSF stroke volume between 21 RRMS patients and 20 HCs [6]. Albeit it did not survive statistical multiple comparison correction, the increase in AoS CSA was accompanied by expected increase in CSF flow rate (p = 0.023 for diastolic flow rate peak and p = 0.051 for systolic flow rate peak).

On the other hand, we did demonstrate significant enlargement of the AoS, a finding which was particularly evident within the progressive MS patients. Moreover, the multiple regression analysis highlighted the relationship between longitudinal change in the size of AoS and global atrophy as measured by expansion of the total vCSF space and local changes as measured by thalamic atrophy. Furthermore, the statistical findings in this particular regression model did not include the baseline or the longitudinal change within the maximal velocity as a contributing factor that was associated with the change in AoS CSA. Therefore, these findings can potentially argue against internal fluid dynamics within the CSF space, but rather a passive dilatation of the AoS due to MS-driven neurodegenerative changes. Central brain atrophy measured as expansion of the third ventricle and/or as expansion of the lateral ventricles is one of the most reliable neurodegenerative MS biomarkers which have been strongly associated with clinical disability and disease progression [28]. The greater enlargement of the AoS within the PMS group is in line with the increasing MS literature that shows accelerated atrophy rate in these later stages of the disease [29]. Furthermore, recent findings have shown that the expansion of the ventricles may be due to atrophying periventricular lesions [30]. The chronic inflammatory processes that persist within these periventricular lesions would lead to absolute destruction of the brain tissue and its transformation into expanded CSF spaces [30]. An example of greater ventricular enlargement in an MS patient when compared to HC is shown in Additional file 2: Figure S1. These processes result in a non-uniform/non-symmetric expansion of the ventricles when compared to symmetrical expansion produced by high ventricular pressure and high CSF velocities. These findings are further corroborated by the fact that our PMS patients exhibited both numerically lower T2 lesion volume accrual and greater vCSF expansion rate over the 5-year follow-up period. Moreover, the thalamus, a highly interconnected brain hub, is particularly vulnerable structure affected by lesion-driven retrograde and/or anterograde (Wallerian) neurodegeneration [31]. Since the thalamus provides anatomical borders with the third ventricle and it is in the close proximity to the AoS, atrophic thalamus changes would significantly impact the AoS size.

Outside of the MS field, studies have utilized vascular and CSF dynamics in order to differentiate age-associated neuropathologies and the resulting ventricular expansion [32]. For example, the hydrodynamic abnormalities in amnestic mild cognitive impairment (aMCI) and the subsequent Alzheimer’s disease (AD) may be initiated by changes in arterial flow changes first, whereas the hydrodynamic abnormalities in NPH may originate from changes within the CSF flow [33]. Our CSF hydrodynamic results also fall in line with measures in the aMCI and AD populations. When compared to NPH, MS patients and other neurodegenerative diseases display 4 to 5 times lower aqueductal stroke volume (30–40 µl/beat in MS/AD/aMCI vs. 160–170 µl/beat in NPH) [34]. Furthermore, the expansion in AoS CSA and ventricular volume seen NPH patients is associated with this aforementioned aqueductal stroke volume, whereas the AoS CSA change in the classical neurodegenerative diseases may be driven by extraventricular factors [34]. Lastly, the absolute stroke volume does increase with the normal processes of aging (33% for every following year) and differs between sexes (20% higher in males) [35]. Although we did statistically adjust for both demographic covariates, the small increase in systolic and diastolic flow rate seen in our MS population can be potentially explained by intensified aging brain interactions [35].

A limitation to our work is the relatively small number of MS patients and HCs. Further analysis between longitudinal changes in CSF measures and clinical disability accrual/phenotype progression should be considered. Also, future studies should include multiple PC sequences with different velocity encodings which would allow simultaneous examination of the changes within CSF fluid dynamics together with hemodynamic measures of the arterial and venous counterparts. Changes in AoS velocity, pressure gradients, and flow measures due to heart cycle changes, respiration or exercise should be monitored in future studies [36]. As such, a recent study demonstrated that respiratory-induced pressure gradients—the force driving the net CSF flow through the AoS—dominate over the cardiac component which is responsible for more frequent and pulsatile peak flow rates [37]. That being said, the PC-MRI measurements may mostly depict the short-term pressure fluctuations and poorly correlate with the overall intracranial pressure changes [38]. Lastly, attempts at MRI analysis of newly demonstrated cerebral glymphatic and lymphatic flow can contribute towards more comprehensive understanding of the brain fluid dynamics [39]. If brain fluid dynamics contribute towards the multifaceted pathophysiological MS mechanisms, either pharmacological or rehabilitative treatments may alleviate some the aforementioned abnormalities [40].

## Conclusions

MS patients present with significant longitudinal AoS area dilatation, potentially due to regional atrophy changes and ex-vacuo expansion of the aqueduct. There were no significant changes in velocity and flow dynamics of the CSF measures. MS brain atrophy may be responsible for a significant part of the functional CSF changes previously seen in the MS population.

## Supplementary information

**Additional file 1: Table S1.** Longitudinal analysis of phase contrast flow measures in RRMS and PMS subpopulations. **Table S2.** Comparison of phase contrast flow measures between RRMS and PMS patients.

**Additional file 2: Figure S1.** Ventricular enlargement example in MS patients when compared to HC. *MS* multiple sclerosis, *HC* healthy control, *vCSF* ventricular cerebrospinal fluid, A—MS patient demonstrating significant 80% enlargement of the ventricular CSF spaces. Note the ventricular expansion and the significant atrophy of the thalamus. B—healthy control demonstrating low rate of vCSF expansion over the same follow-up period.

## Data Availability

The datasets used and/or analyzed during the current study are available from the corresponding author on reasonable request.

## Abbreviations

MS: multiple sclerosis
CNS: central nervous system
WM: white matter
CSF: cerebrospinal fluid
MRI: magnetic resonance imaging
PC: phase contrast
HCs: healthy controls
AoS: Aqueduct of Sylvius
EDSS: Expanded Disability Status Scale
RRMS: relapsing-remitting multiple sclerosis
PMS: progressive multiple sclerosis
TE: echo time
TR: repetition time
TI: inversion time
FLIP: flip angle
FOV: field of view
CSA: cross-sectional area
WBV: whole brain volume
vCSF: ventricular cerebrospinal fluid
ANCOVA: analysis of covariance
FDR: false discovery rate
GLM: general linear model
DMT: disease modifying treatment
IR-FSPGR: spoiled gradient echo with inversion recovery preparation
Vmean: average velocity
Vmax: maximal velocity
BL: baseline
FU: follow-up
