# Supplementary material for: Longitudinal analysis of cerebral aqueduct flow measures: multiple sclerosis flow changes driven by brain atrophy
Source: Fluids Barriers CNS. 2020 Jan 31;17:9. doi: 10.1186/s12987-020-0172-3 (PMC6993504; doi:10.1186/s12987-020-0172-3)
Supplement: Supplementary file 1 — Additional file 1: Table S1. Longitudinal analysis of phase contrast flow measures in RRMS and PMS subpopulations. Table S2. Comparison of phase contrast flow measures between RRMS and PMS patients. [file 12987_2020_172_MOESM1_ESM.doc]

**Table S1.** Longitudinal analysis of phase contrast flow measures in RRMS and PMS subpopulations

|  | RRMS (n=15) | | | PMS (n=14) | | |
| --- | --- | --- | --- | --- | --- | --- |
| AoS-derived measures | Baseline | Follow-up | BL to FU p-value | Baseline | Follow-up | BL to FU p-value |
| Vmean systolic peak (cm/s) | 5.38±2.59 | 5.55±1.78 | 0.77 | 5.54±1.67 | 5.31±1.74 | 0.525 |
| Vmean diastolic peak (cm/s) | -4.22±1.69 | -4.32±1.31 | 0.718 | -4.21±0.84 | -3.78±1.04 | 0.128 |
| Vmax systolic peak (cm/s) | 10.1±3.9 | 9.69±3.14 | 0.626 | 9.58±3.27 | 9.37±2.89 | 0.708 |
| Vmax diastolic peak (cm/s) | -7.13±2.17 | -8.03±2.73 | *0.053* | -7.33±1.88 | -7.69±2.89 | 0.386 |
| Average Area (mm2) | 3.02±0.89 | 3.44±1.31 | *0.078* | 3.24±1.27 | 3.96±1.78 | **0.004*** |
| Flow rate systolic peak (ml/min) | 9.5±4.51 | 11.8±7.34 | 0.16 | 11.27±6.5 | 12.54±7.51 | 0.144 |
| Flow rate diastolic peak (ml/min) | -7.41±3.2 | -9.35±5.85 | 0.113 | -8.14±3.9 | -9.31±5.62 | *0.075* |
| Net vol caudal (µl/beat) | 42.56±23.42 | 50.21±37.35 | 0.298 | 51.61±31.15 | 50.78±32.34 | 0.826 |
| Net vol cranial (µl/beat) | -36.96±21.01 | -46.26±35.71 | 0.167 | -45.6±26.05 | -45.44±29.37 | 0.959 |

**Legend:** RRMS – relapsing-remitting multiple sclerosis, PMS – progressive multiple sclerosis, AoS – Aqueduct of Sylvius, Vmean – average velocity, Vmax – maximal velocity, BL – baseline, FU – follow-up.

All measures are shown as mean ± standard deviation. Paired repeated measure analysis was utilized. P-values lower than 0.05 were considered statistically significant and shown in bold where trending values are shown in italics. * - statistically significant after false discovery rate correction.

**Table S2.** Comparison of phase contrast flow measures between RRMS and PMS patients

|  | At baseline | | | At follow-up | | |
| --- | --- | --- | --- | --- | --- | --- |
| AoS-derived measures | RRMS (n=21) | PMS (n=14) | RRMS vs PMS | RRMS (n=17) | PMS (n=14) | RRMS vs PMS |
| Vmean systolic peak (cm/s) | 5.47±2.24 | 6.12±2.53 | 0.394 | 5.48±1.68 | 5.31±1.74 | 0.692 |
| Vmean diastolic peak (cm/s) | -4.18±1.57 | -4.49±1.27 | 0.464 | -4.34±1.23 | -3.78±1.04 | 0.432 |
| Vmax systolic peak (cm/s) | 9.81±3.52 | 9.82±3.55 | 0.95 | 9.65±2.94 | 9.37±2.89 | 0.771 |
| Vmax diastolic peak (cm/s) | -7.2±2.01 | -7.5±2.35 | 0.664 | -7.92±2.57 | -7.69±2.89 | 0.893 |
| Average Area (mm2) | 3.03±0.98 | 3.3±1.46 | 0.707 | 3.27±1.31 | 3.96±1.78 | 0.393 |
| Flow rate systolic peak (ml/min) | 9.73±4.82 | 11.92±6.13 | 0.259 | 11.11±7.14 | 12.54±7.51 | 0.714 |
| Flow rate diastolic peak (ml/min) | -7.43±3.23 | -8.66±4.05 | 0.361 | -8.92±5.61 | -9.31±5.62 | 0.978 |
| Net vol caudal (µl/beat) | 44.48±25.57 | 51.82±27.54 | 0.444 | 47.99±35.6 | 50.78±32.34 | 0.855 |
| Net vol cranial (µl/beat) | -36.52±21.22 | -46.98±25.85 | 0.367 | -43.88±34.19 | -45.44±29.37 | 0.902 |

**Legend:** RRMS – relapsing-remitting multiple sclerosis, PMS – progressive multiple sclerosis, AoS – Aqueduct of Sylvius, Vmean – average velocity, Vmax – maximal velocity.

All measures are shown as mean ± standard deviation. Analysis of covariance (ANCOVA) adjusted for age and sex was utilized. P-values lower than 0.05 were considered statistically significant and shown in bold where trending values are shown in italics.
