# Supplementary figures and images for: Longitudinal analysis of cerebral aqueduct flow measures: multiple sclerosis flow changes driven by brain atrophy
Source: Fluids Barriers CNS. 2020 Jan 31;17:9. doi: 10.1186/s12987-020-0172-3 (PMC6993504; doi:10.1186/s12987-020-0172-3)

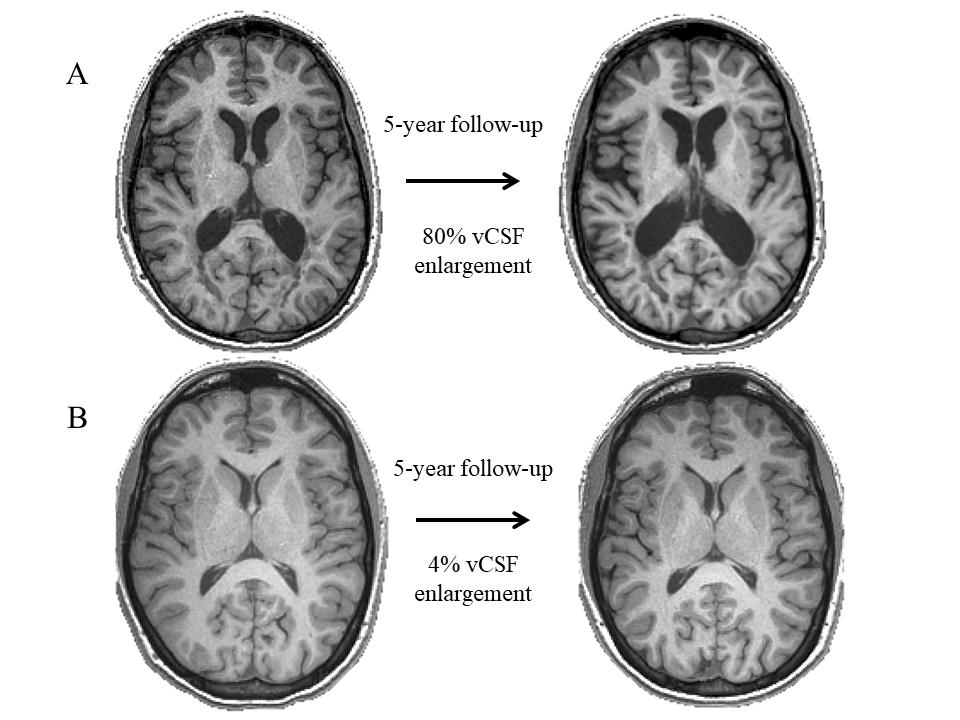

Supplement: Supplementary file 2 — Additional file 2: Figure S1. Ventricular enlargement example in MS patients when compared to HC. MS multiple sclerosis, HC healthy control, vCSF ventricular cerebrospinal fluid, A—MS patient demonstrating significant 80% enlargement of the ventricular CSF spaces. Note the ventricular expansion and the significant atrophy of the thalamus. B—healthy control demonstrating low rate of vCSF expansion over the same follow-up period. [file 12987_2020_172_MOESM2_ESM.tif]
